# Supplementary material for: Scaffolding proteins guide the evolution of algal light harvesting antennas
Source: Nat Commun. 2021 Mar 25;12:1890. doi: 10.1038/s41467-021-22128-w (PMC7994580; doi:10.1038/s41467-021-22128-w)
Supplement: Supplementary file 1 — Supplementary Information [file 41467_2021_22128_MOESM1_ESM.pdf]

Supplementary Information

Scaffolding proteins guide the evolution of algal light harvesting antennas

Authors: Harry W. Rathbone[1], Katharine A. Michie[1,2], Michael J. Landsberg[3], Beverley R. Green[4], Paul M. G. Curmi[1*.]

[1] School of Physics, University of New South Wales, Sydney, NSW 2052, Australia.

[2] Mark Wainwright Analytical Centre, University of New South Wales, Sydney, NSW 2052, Australia.

[3] School of Chemistry and Molecular Biosciences, The University of Queensland, St. Lucia, QLD, Australia.

[4] Botany Department, University of British Columbia, Vancouver, BC V6N 3T7, Canada.

*Correspondence to: p.curmi@unsw.edu.au.

Supplementary Figures 1 - 9
Supplementary Tables 1 - 2
Supplementary Notes 1 – 14
Supplementary References

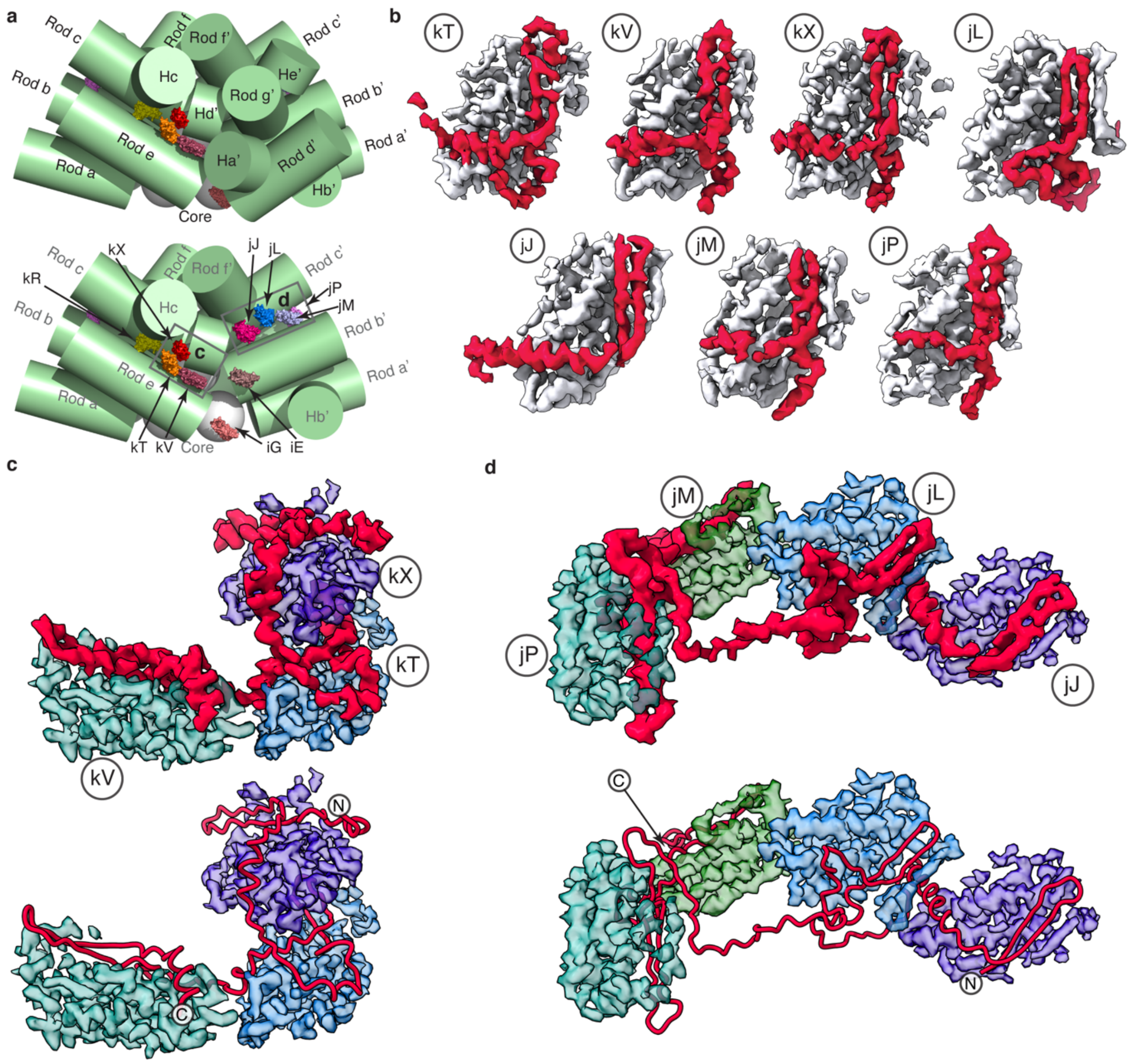

Supplementary Figure 1. CALM domain proteins stabilise the isolated PE β subunits in the PBS of the red alga *G. pacifica*. **a**. Cartoon representation of the cryo-EM structure of the PBS from *G. pacifica* (PDB:5Y6P) [1] shows the presence of 'lone' PE β subunits (coloured molecular surface rendering) that are peripherally associated with the PBS rod structures (green cylinders). The lower image is a cut away, where Rod g', Rod d' and hexamer rings: Hc, Hd', Ha' and He' have been removed to reveal the isolated PE β subunits that are buried in the PBS. The core of the PBS here sits directly above the thylakoid membrane (bottom). The boxed subunits correspond to clusters detailed in **c** and **d**, respectively. **b**. Sculpted EM map density showing the individual CALM domains (red) and their associated PE β subunits (grey). The lone PE β subunits in *G. pacifica* are arranged in two clusters of three and four subunits. Each cluster is associated with a single CaRSP with three and four CALM domains, respectively. **c**. Shows sculpted EM map density (upper panel) for the three-CALM CaRSP (red) with its associated PE β subunits (kX - violet, kT - blue and kV - teal), while **d**. shows the sculpted EM map density (upper panel) for the four-CALM CaRSP1 (red) with its associated PE β subunits (jJ - violet, jL - blue, jP - teal and jM - green). The lower panels in **c** and **d** show the tube trace of the CaRSP backbone (red) with N- and C-termini labelled.

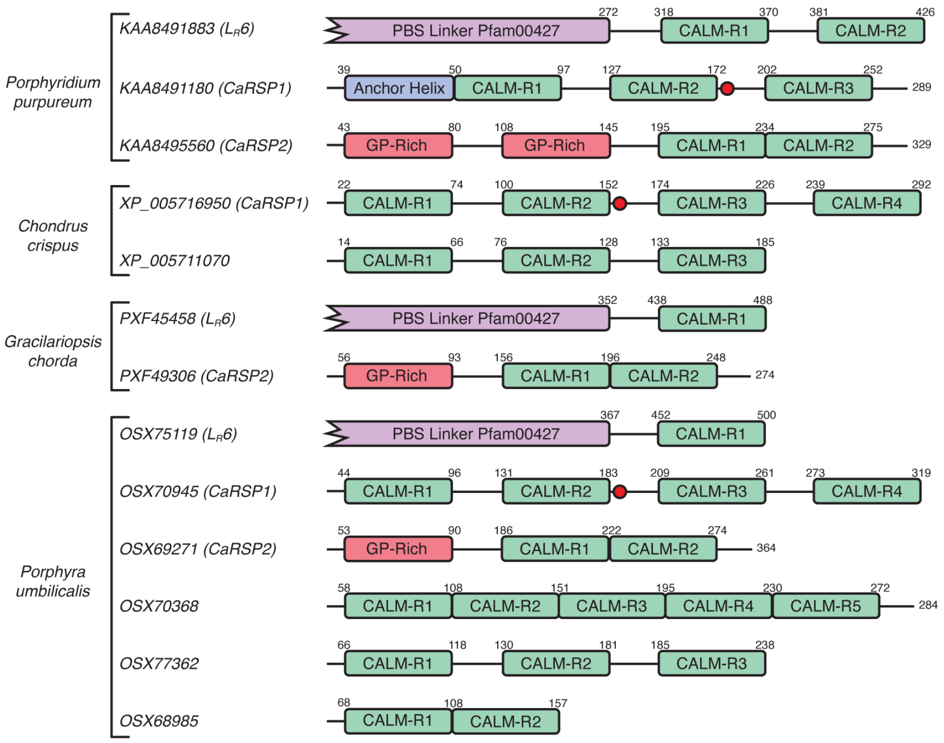

Supplementary Figure 2. Domain organization of the red algal CaRSPs. The 13 red algal CaRSPs have been ordered by organism with CALM domains shown as green rectangles. There are three members of the CaRSP1 family (*P. purpureum KAA8491180*, *C. crispus XP_005716950* and *P. umbilicalis OSX70945*) which contain three or four CALM domains with a signature F-W-K-[TS] motif between the second and third CALM domains (signified by the red dot). This aromatic motif serves to anchor the CaRSP against PBS rod structures (see Supplementary Fig. 4d-f). There are three CaRSP2 family members (*P. purpureum KAA8495560*, *G. chorda PXF49306* and *P. umbilicalis OSX69271*) that share an N-terminal Gly-Pro rich motif (pale red rectangle). There are three L_R6 ("Linker 2" [2]) family members (*P. purpureum KAA8491883*, *G. chorda PXF45458* and *P. umbilicalis OSX75119*) that share an N-terminal PBS linker (Pfam00427) domain (purple rectangle) followed by one or two CALM domains. Four CaRSPs remain unclassified. Lengths of each domain (as shown by protein sequence numbers at the beginning and end of each domain) should be taken as an illustrative guide. As a general rule the start of the CALM domain was taken to be 10 residues before the N-X-A-P motif and approximately 52 residues long (with exceptions). The terminus of Pfam00427 was chosen from a conserved G-L-P sequence.

```
                      10         20         30         40         50         60         70         80
                       |          |          |          |          |          |          |          |
PE545 α1    -----AMDKS AKAPQITIFD HR-GC-SRAP KESTGGKA-G GQDDEMMVKV ASTKVTVSES DAAKKLQEFI TFEKGIDGPF TSKN-----
PE545 α2    -----AMDKS AKAPVITIFD HR-GC-SRAP KEYTGAKA-G GKDDEMMVKA QSVKIEVSTG TAEGVLATSL AKMTK----- ---------
PC645 α1    ------RDAQ LRAPIVEIFD AR-GC-DAKN AQYTGPKS-N DMNDDQCVKV SMQKITVSEA TAAKKLQEFI GGKATAINVP IISSMTKKY
PC645 α2    ------KDAQ LRAPVVTIFD AR-GCKDHAN KEYTGPKAGN AENDECCVKV QMTPIKVADD AAALVLKECL SELKGKK--- ---------
PE555 α1    -----AMKKD SKAPCVEVFD ERDGCKAAGT QKAS------ -GDDGFCVKV SMKAIGFNAA EAASVTKNYG IKRFGAKSV- ---------
PE555 α2    -----AMKKD SKAPCVEVFD ERDGCKAAGT QKAS------ -GDDGFCVKV SMKAIKMNAA EATSVTKNYN TKLL------ ---------
PC612 α     -----KMATD SKAPLIELFD ERDGCKGPAA NKASDV---- -GEPGLCVKV SMQKVAMNAA AAKSVATNYM RK-------- ---------
Pp 1180 R1  TAKKNQYMGT SVAPEIVLTD KGSDMS---- ---------- -----RKVKT EDKKVAADQA AAMGILANMS L--------- ---------
Pp 1180 R2  VEDFYPSSWR NMAPVISLSA ---------- ---------- -----NRVAV AFEKIDAASN GVKANSNNKP -FWKSGAT-- ---------
Pp 1180 R3  YQRYFPARIR NKAPAMEFRR PSFANTEDPS ---------- -----AYFML QKETVPLRMA LAEKLLT--- ---------- ---------
Pp 1883 R1  YTSLCSYDKV NKAPQISVTN VGSDEH---- ---------- -----SYVSV TSKYIAPDMA AAAQMLADCQ K--------- ---------
Pp 1883 R2  WMKYYPGTTV NMAPYISLND TGSDSS---- ---------- -----RTVSV TLDKVKISPN KIGK------ ---------- ---------
Pp 5560 R1  YNKYFSADRL HKAPEILFEY NKTKY----- ---------- -----DRVGV RYTEVTSKA- ---------- ---------- ---------
Pp 5560 R2  SERFFPKSRM NRAPVIEISY REGAVST--- ---------- -----ASVSL SMPEISGPPA LPFPVPKGDV ---------- ---------
Cc 1070 R1  YLSFIPVDRL NKAPVITINN FSNEQV---- ---------- -----NSVSV RLADFFRDEG AGNSVKAGVK I--------- ---------
Cc 1070 R2  WAKYYDPQTV NEAPIISIYS SVDDLPYTK- ---------- -----WGVSI IQTVVPTRLD EGRKLLGD-- ---------- ---------
Cc 1070 R3  DSYKEYIPNT NMAPDITVSL GATDEQ---- ---------- -----SNVTV DMNTQFTSEA AVEAIFAQFR R--------- ---------
Cc 6950 R1  HRTPRCSMGD SIAPKIVVVN TGNEQT---- ---------- -----SRLTL EWAAIPPKTG DATSVLANTA LK-------- ---------
Cc 6950 R2  KNFYFPPETR NMAPVISMTA N--------- ---------- -----ESISV AVESINPSVA NVKANSPDSV AFWKTP---- ---------
Cc 6950 R3  YEAYFPTKIR NLAPKISMRP PAGDWDKT-- ---------- -----AYLIL DKEFVALNPD LARQLTVKP- ---------- ---------
Cc 6950 R4  VEKFFGGEFM SKAPQISIDQ DI-------- ---------- -----PKVEF SLGDISPSAE TVSAVFAEVG RPEGDQ---- ---------
Gc 5458 R1  KNYVGSRKTF DMAPVIELRP PTSAIAQN-- ---------- -----GVLSV SMQKITFPVP SEMQQAV--- ---------- ---------
Gc 9306 R1  YEKYFPDSVR NKAPEIKIVY ENDYLK---- ---------- -----DRVSV AMTEVTASF- ---------- ---------- ---------
Gc 9306 R2  SDVYYPKEMK NKAPVIDISY NGNLAT---- ---------- -----ASVSV SMEHIEGLPT LPPPPKAEGD T--------- ---------
Pu 5119 R1  SYTGGARKVW DKAPMVELKT PKNGQG---- ---------- -----GYVRV SLETVTGSIP SNLSQKV--- ---------- ---------
Pu 7362 R1  YSFRFPPSRL NRAPFIVINN IGSSDET--- ---------- -----RSVIV SNVEMRTDIG AGDSLKAGVT ---------- ---------
Pu 7362 R2  VARFYP-SGM NVAPFVSVHD TKNPYT---- ---------- -----QSVSV VMSEVPVNLR SGAAILGGDS E--------- ---------
Pu 7362 R3  ALTTFAGRGL NQAPNVTATY RTSNDD---- ---------- -----SLVYM DKTEVPLPMA EAAAILAKYR KN-------- ---------
Pu 9271 R1  YEQFYPADKR YLAPHIAISE AN-------- ---------- -----DRVGL SMNAVEAGF- ---------- ---------- ---------
Pu 9271 R2  YERYFPKERL NKAPVIDIFY SGSLNT---- ---------- -----ASVSL KMEEVEGLPA VAVVPKGDVQ T--------- ---------
Pu 0368 R1  YQQYYP-GPL HRAPHIVFQR SGE------- ---------- -----DAVIV DMAEVPLPDT HGGADPDTPN VAA------- ---------
Pu 0368 R2  YADHFPAAGL HMAPHITIDG S--------- ---------- -----EGVGL EMKTVDTPVA PALSPAL--- ---------- ---------
Pu 0368 R3  YNKFFPEDRL HMAPHISFSD K--------- ---------- -----SVIKV QMRAVATDVE GADRPPFE-- ---------- ---------
Pu 0368 R4  AAEYGKYGGL NWAPVITLTD GGADK----- ---------- -----QGVRV AMEAI----- ---------- ---------- ---------
Pu 0368 R5  ----SGDGKL NAAPVILFDG T--------- ---------- -----SRVGL TMPLIEGKAA ADEAAATAAA VTYTPG---- ---------
Pu 0945 R1  SRDPAAARPK DVAPMITLSF AQPASDLDGT ---------- -----AKIGL SMAPVGLDTA TAQTILA--- ---------- ---------
Pu 0945 R2  KDVYFPAATR NQAPVISFGA AN-------- ---------- -----DSISV AYTAINPVVG GQAANSSDSV AFWKT----- ---------
Pu 0945 R3  YERYFPTKIR NRAPVISMRP PAGPWDTT-- ---------- -----AYLTV GSEVVALNSS AARQVSVET- ---------- ---------
Pu 0945 R4  VQKNFGGDRA NKAPVIAIGE ---------- ---------- -----ESVTL SMEEVAVGED AAASVSSAAS A--------- ---------
Pu 8985 R1  VDAAFPPPTR GLAPDISFAA GT-------- ---------- -----LGVSV AMTRATGGVP PPD------- ---------- ---------
Pu 8985 R2  AYARFAASGL HRAPVIELKA PAGGWDTVG- ---------- -----GYVTV GSEEVPLRLD VAARL----- ---------- ---------

Motif       ΩxxΩΩP     NxAPxIxϕ                                  VxV xMxxϕ                        FWKT
```

Supplementary Figure 3. Sequence alignment of CALM motifs. Sequence alignment of all 34 CALM motifs from the 13 CaRSPs along with the cryptophyte α subunits whose structures have been determined with structure-based alignment of cryptophyte α subunits and *P. purpureum* CALMs as a reference alignment. The CALM motif is shown at the bottom of the alignment including the F-W-K-[TS] motif (violet) that is unique to the second CALM domain in CaRSP1. Lengths of each domain (shown in Supplementary Fig. 2) should be taken as an illustrative guide. In the motif, Ω represents Y/F/W and ϕ represents hydrophobic residues. Each CALM domain is given the name based on the initials of their species, the last four numbers from the protein accession code (as per Supplementary Fig. 2) and the CALM repeat (R1, R2, R3, R4 and R5). Residue colouring indicates: red – strictly conserved; blue – similar; orange – aromatic motif; violet – FWKT motif; and green – chromophore interacting (cryptophyte α subunits only).

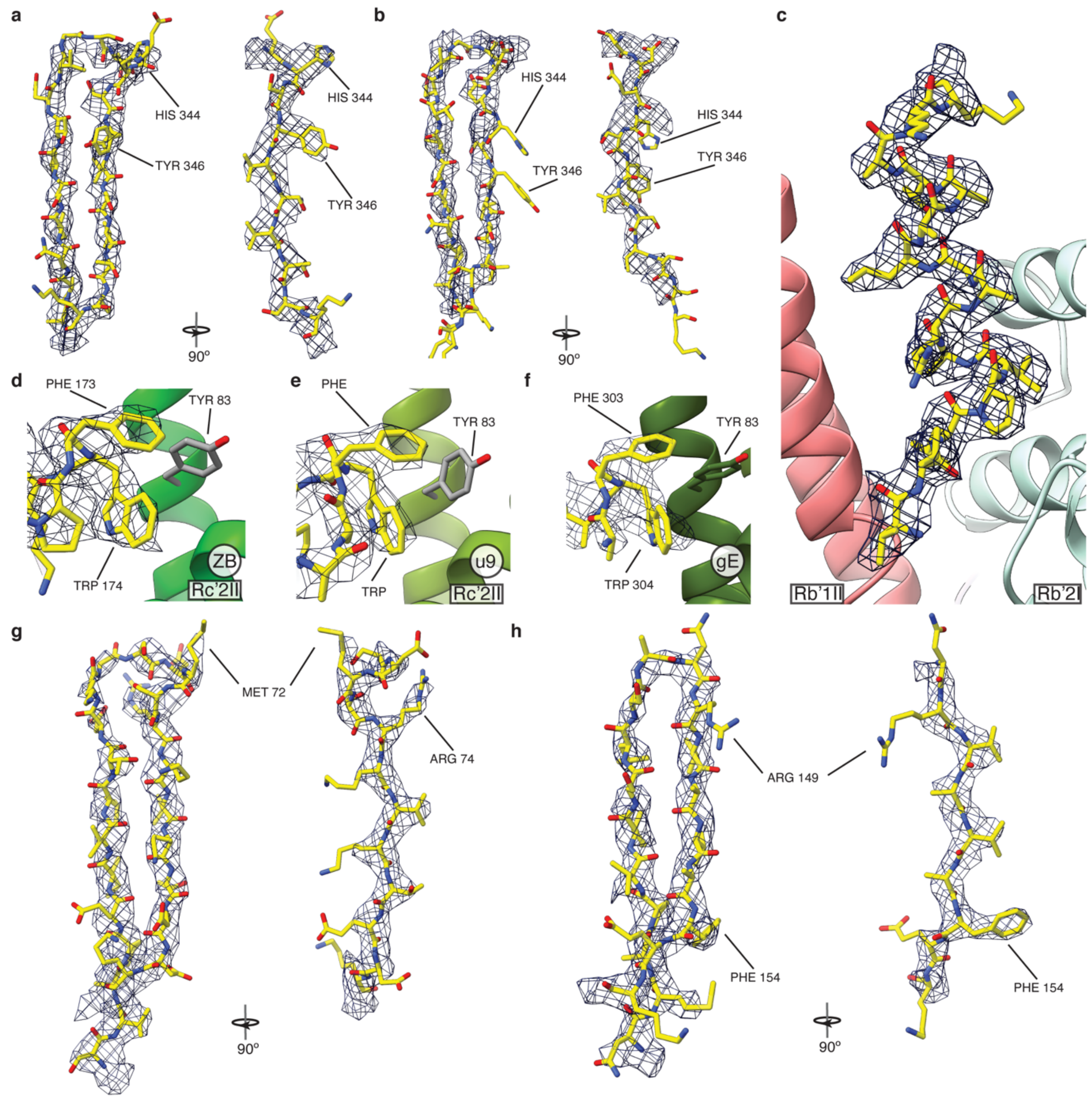

Supplementary Figure 4. Structural elements of the CALM:PE β complexes a. The refined model for the first CALM domain of rod linker L_R6 in two orientations. **b** The original model (as per Ma *et al.* 2020 [3]) for CALM1 of L_R6 in the same orientations. In each of these panels, the two views shown are related by a 90° rotation about the y-axis. The first view is "face-on" when looking at the CALM:PE β complex and the second view shows only the exiting β strand S2 of the β ribbon as seen from the entering strand S1. **c**. The EM map density for the N-terminal anchoring helix of CaRSP1 from *P. purpureum*. This helix penetrates between the PBS trimers Rb'1II and Rb'2I (shown in pale red and pale blue). **d.** and **e**. The F-W-K-T motif between CALM domains two and three in CaRSP1 for *P. purpureum* and *G. pacifica*, respectively, where the model for *G. pacifica* was polyalanine, except for this motif. This motif docks to the PBS rod structure trimer Rc'2II via helix hE of the PBS phycoerythrin α subunit interacting with Tyr83 (green cartoon representation showing Tyr83, only). **f.** The same structural motif as panels **d.** and **e** from the model for the C-terminal tail of "Linker 3" from *P. purpureum*. **g.** and **h**. The first two CALM domains of CaRSP1 respectively, showing two views with the first as per panels **a** and **b** and second view showing the strand S2 only with a 90° rotation between the two views (as per the rotation marker in the panel). Residues are marked as a reference to frame sequence. **g.** shows

a helical turn between the two β strands (top right of first view) and **h**. shows the tight turn (top of first view). All panels show cryo-EM density for *P. purpureum* (EMD-9976) except for panel **e**, which shows *G. pacifica* (EMD-6769).

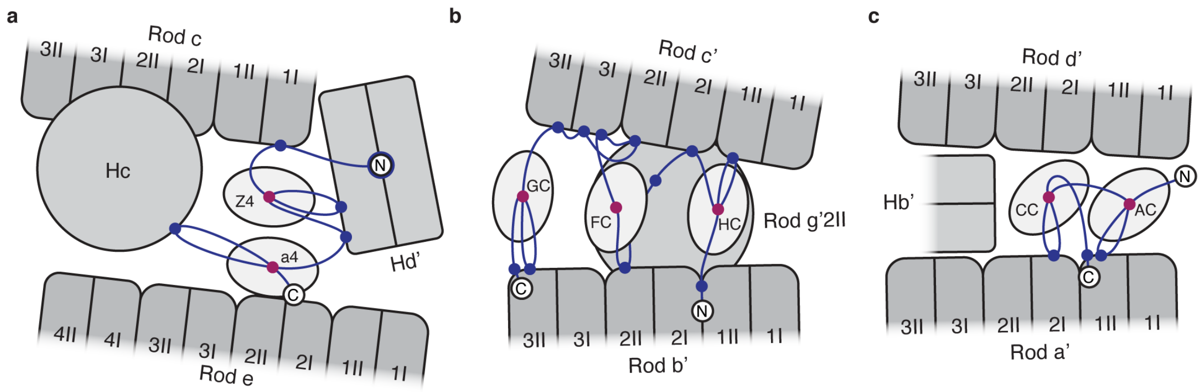

Supplementary Figure 5. Diagrammatic representation of contacts made by each CaRSP to PBS rods, hexamers and CALM-bound PE β subunits. Each PBS $(\alpha\beta)_3$ trimer is labelled. **a.** L_R6 **b.** CaRSP1 **c.** CaRSP2. Each blue dot represents a contact to a PBS rod or hexamer and each red dot represents a CALM domain. Where a loop is formed from a red dot to a contact site (blue dot), the blue dot corresponds to the turn between β-strands of that particular CALM domain. Each CaRSP is traced from "N", representing the N-terminus. Contacts between CALM-bound PE β subunits are not shown for simplicity and distances are exaggerated for clarity.

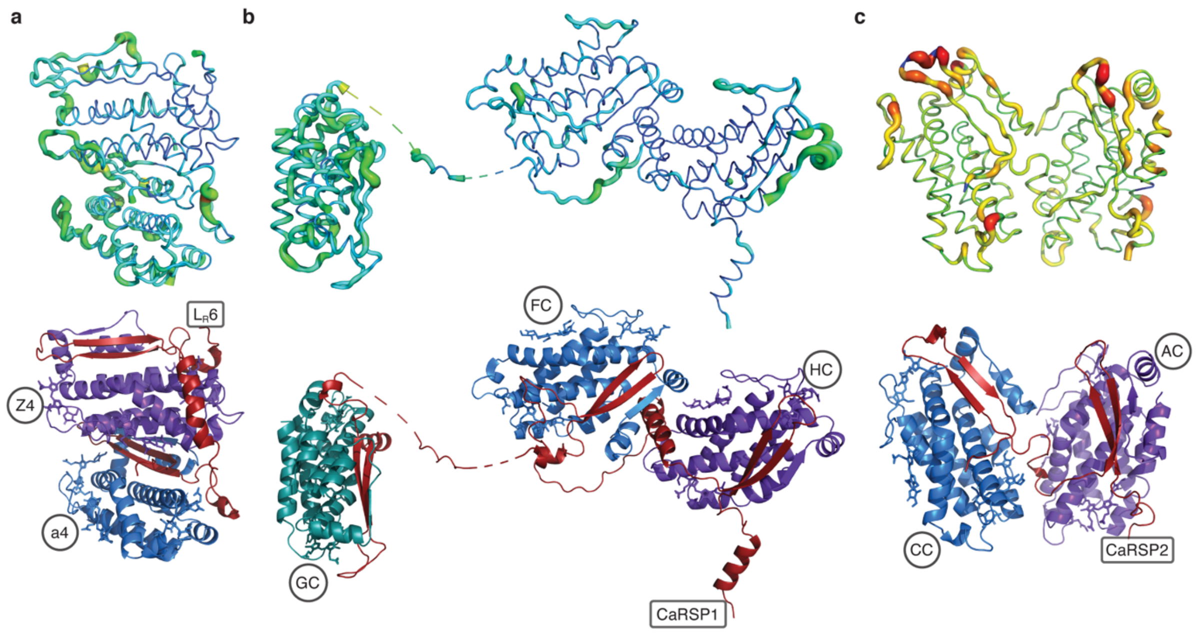

Supplementary Figure 6. Model/map quality characterized by correlation coefficient (CC) between the model and the cryo-EM map. The models are **a.** L_R6 **b.** CaRSP1 **c.** CaRSP2. Top row provides a 'putty' representation for the CC value at each residue, where the colour represents the CC on an absolute scale for all models while the width of the ribbon represents the CC relative to the individual model. The redder (and wider) the ribbon, the lower the CC value and hence the poorer the model and/or map in that region. The bottom row provides a cartoon representation of the structure as a reference. The CaRSPs/L_R6 are shown in red while the PE β subunits are shown in violet, blue and teal.

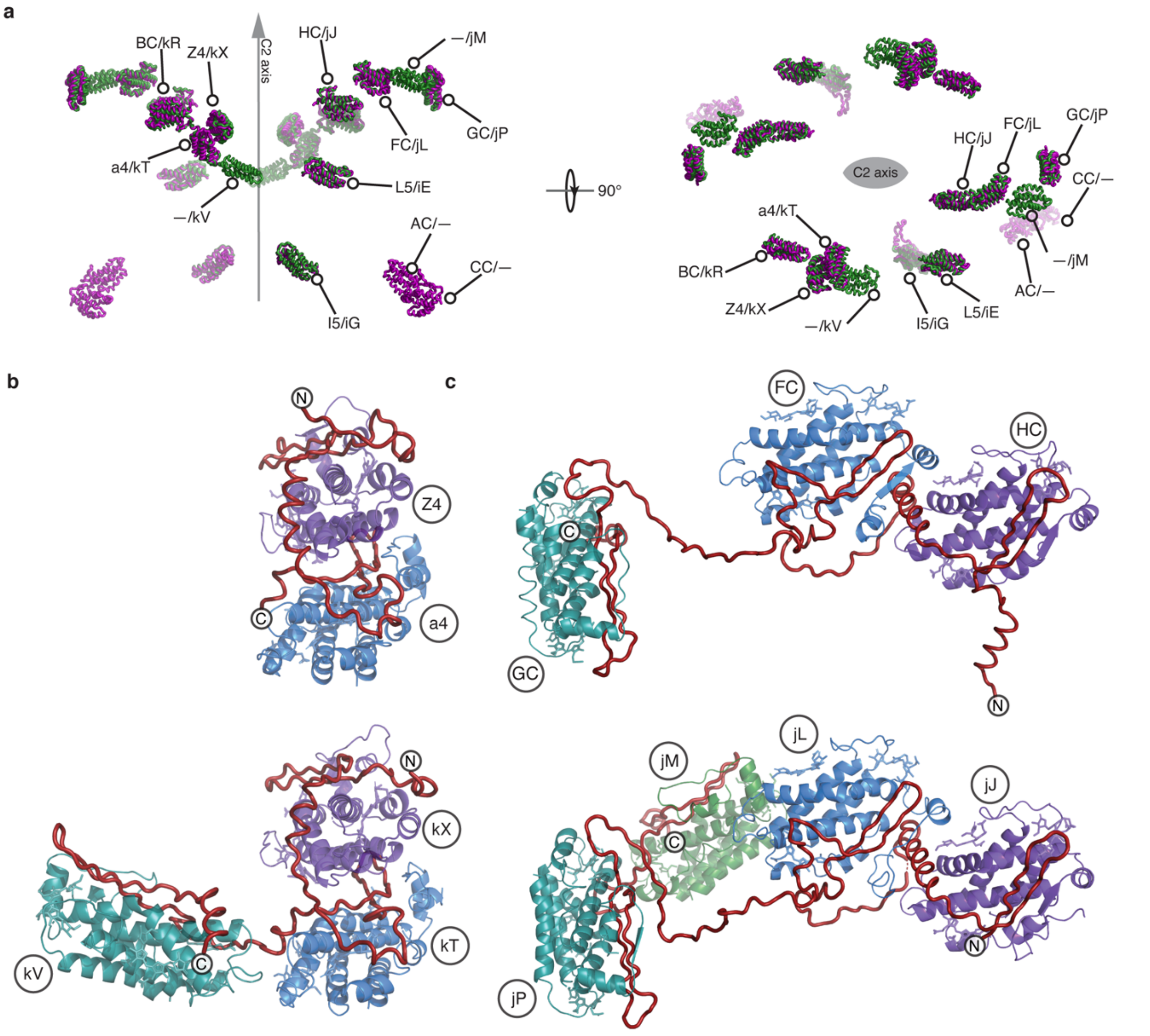

Supplementary Figure 7. Comparison of the arrangement of 'lone' PE β chains and their associated CaRSPs in *G. pacifica* (PDB:5Y6P) and *P. purpureum* (PDB:6KGX). **a**. Overview of the arrangement of 'lone' PE β subunits in *G. pacifica* (green) versus *P. purpureum* (purple). Left panel shows the same orientation as per Fig. 1a or Supplementary Fig. 1a, where the thylakoid membrane is at the bottom with the two-fold symmetry axis perpendicular to the membrane and in the centre of the panel. The right-hand panel is the view down the symmetry axis towards the membrane. The two-fold symmetry axis is represented by the lozenge in each panel. **b**. Top image shows the two PE β subunits (violet and blue) attached to the two CALM domains in rod linker L_R6 (red tube representation). Bottom image shows the three PE β subunits in *G. pacifica* (violet, blue and teal) associated with the three CALM domain CaRSP, where the first two CALM:PE β complexes are precisely located in the same positions as the two from *P. purpureum* attached to L_R6 (RMSD 1.839 Å over 2,257 atoms). **c**. Top panel shows the three PE β subunits (violet, blue and teal) attached to *P. purpureum* CaRSP1 (red tube representation). The lower panel shows the four PE β subunits (violet, blue, teal and green) attached to the four CALM CaRSP1 from *G. pacifica*. Three of the PE β subunits have identical locations in the two PBS (RMSD 1.872 Å over 3,283 atoms).

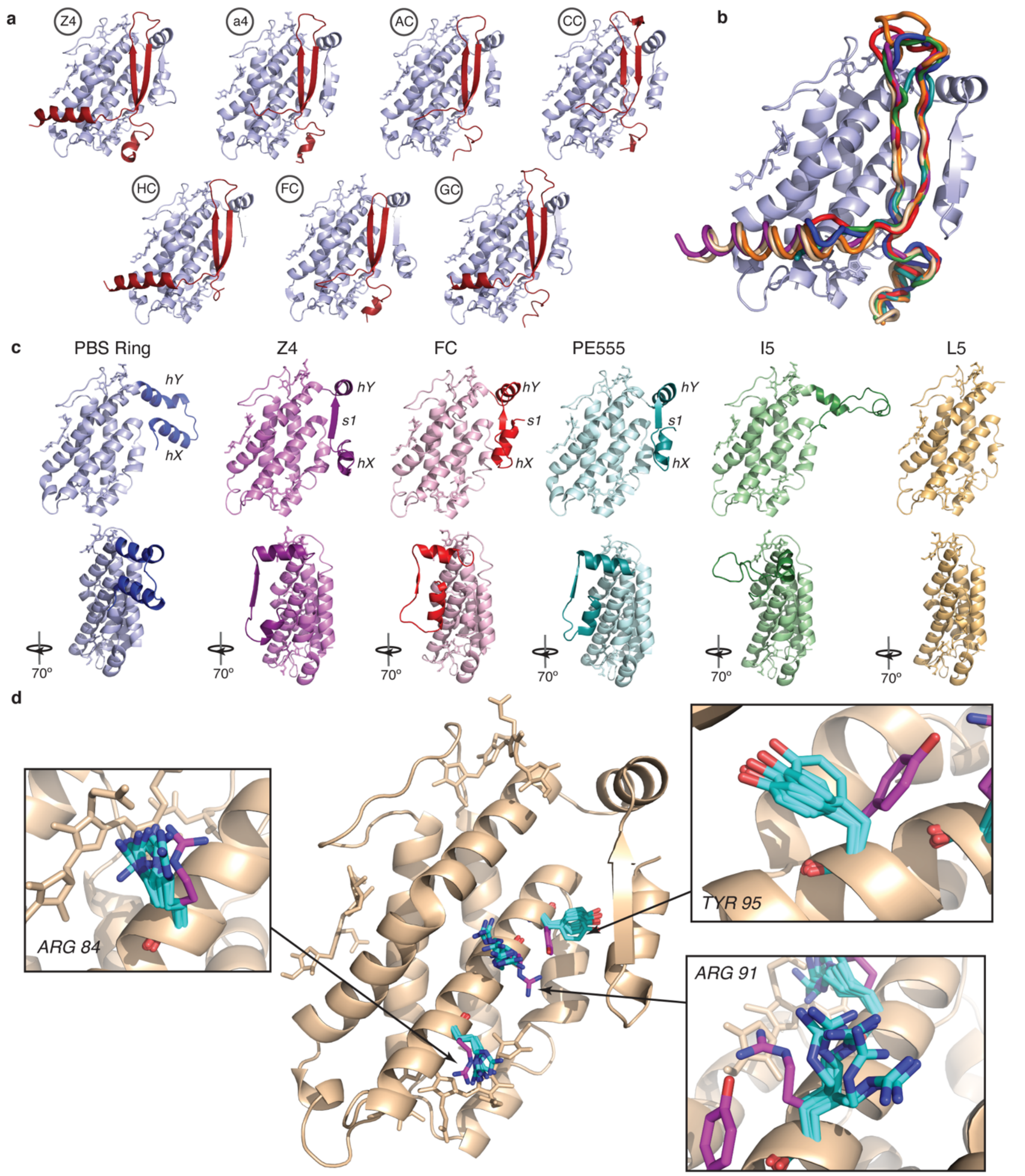

Supplementary Figure 8. Structures of the CALM:PE β complexes in *P. purpureum* and the metamorphic nature of the PE β subunit and scaffolding of non-CALM PE β subunits a. Cartoon representations of the seven unique CALM:PE β complexes (labelled as in Ma *et al.* 2020 [3]), with the CALM domain shown in red. **b**. Structural superposition of all seven unique CALM domains from *P. purpureum*, the colours of which are: tan (L_R6, CALM1, PE β Z4), blue (L_R6, CALM2, PE β a4), purple (CaRSP1, CALM1, PE β HC), teal (CaRSP1, CALM2, PE β FC), orange (CaRSP1, CALM3, PE β GC), green (CaRSP2, CALM1, PE β AC) and red (CaRSP2, CALM2, PE β CC). **c**. Six PE β subunits showing that the globin fold remains invariant, while the N-terminal domain changes with context. Two views are shown where the bottom row is rotated by -70° about the y-axis. Far left image (blue) is the PBS ring complex β subunit from *P. purpureum* showing the N-terminal helices hX and hY forming a helical hairpin projecting from the globin domain. CALM-bound PE β

subunits in *P. purpureum* Z4 and FC (purple and red, respectively) show the rearrangement of helices hX and hY congruent to the cryptophyte *H. andersenii* (PDB:4LMX, cyan). Z4 and FC also show the subtle changes in helix hX. Chain I5 (green) shows helices hX and hY adopting an alternate conformation imparted by the binding partner L_{RC}6 while in chain LR (gold) helices hX and hY appear to have been proteolytically cleaved. **d.** Rotamer changes along helix hE of the PE β subunit upon binding of a CALM domain. Rotamers adopted in the PBS ring conformation (PDB: 3V57) are shown in magenta and the set of rotamers adopted by CALM-bound PE β subunits are shown as the distribution of cyan side chains. Insets for the three residues with large rotamer changes are shown in different orientations to the central image to provide a better view for each.

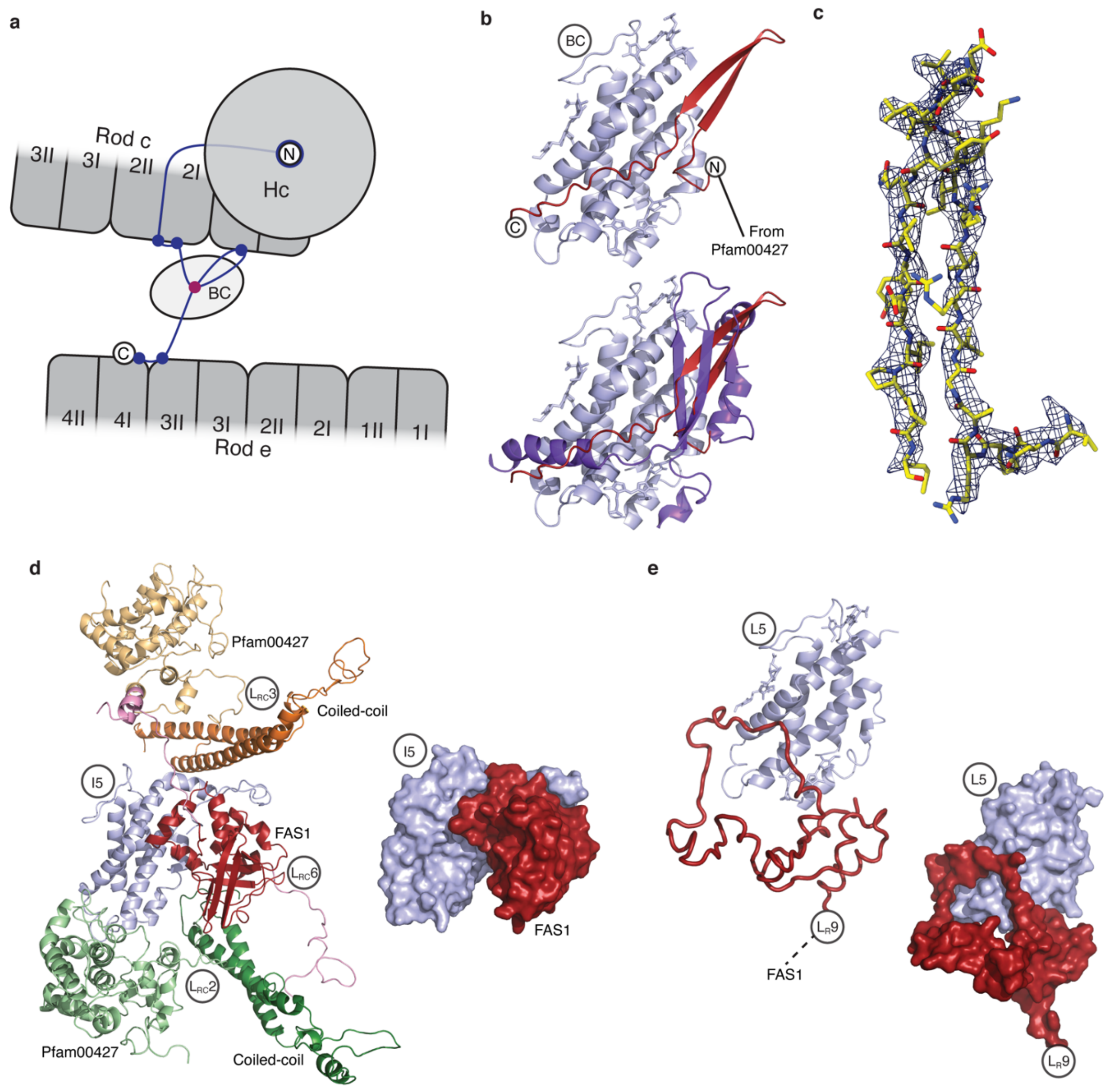

Supplementary Figure 9. The novel PBS linker protein "Linker 3" and the two remaining non-CALM bound 'lone' PE β subunits and their partners. a. The path that can be traced for Linker 3 through electron density showing the contacts made to each rod and the N-terminal Pfam00427 domain in the centre of hexamer Hc. **b**. 'Lone' PE β Chain BC (light blue) bound to the modelled N-terminal β ribbon of "Linker 3" (red) where the C-terminal end makes a contact across the same surface as the helix (or extended chain) of a CALM domain does in the CALM:PE β complexes. The image underneath shows the comparison to the CALM:PE β complex (L_R6:Z4 in purple) as an overlay demonstrating how Linker 3 clashes with the metamorphic helices of PE β and hence the potential cleavage of these helices. **c.** The fit of the twisted β-hairpin of the Linker 3 model to the cryo-EM map. **d**. Outlier PE β subunit, chain I5 (PDB:6KGX, light blue) with its partners: $L_{RC}2$ (green, which contacts the region covered by the helix/turn in CALM domains), $L_{RC}3$ (orange, which glances what remains of helix hY) and $L_{RC}6$ (red, for which the FAS1 domain confers the third metamorphic state of helices hX and hY). The Pfam00427 domains are shown as a lighter colour compared with the coiled-coils of $L_{RC}2$ and $L_{RC}3$ to delimit the two domains. To the right is a molecular surface representation of the PE β:FAS1 complex, where the FAS1 complex binds to the exposed hydrophobic patch of PE β. **e**. Contact of the (possibly)

N-terminally cleaved chain L5 (PDB: 6KGX, light blue) with L_R9 (red). L_R9 makes a contact across the same surface as the helix (or extended chain) of a CALM domain does in the CALM:PE β complexes. The molecular surfaces comprising this complex are shown to the right. Note: for each of these 'lone' PE β subunits (chains: I5, L5) and their symmetry equivalents, the two major hydrophobic regions in PE β are either covered by a partner or (potentially) cleaved off, thus stabilising the protein against aggregation and consequent destruction.

	L_R6:PE β Scaffold<br><br>PDB: *7LIZ*	CaRSP1:PE β Scaffold<br><br>PDB: *7LIX*	CaRSP2:PE β Scaffold<br><br>PDB: *7LIY*	Linker 3:PE β Scaffold<br><br>PDB: *7LJ0*
**Data collection and processing**	***Details can be found in Ma et al.*** [3] ***from which the original maps are published***			
**Refinement**				
Initial model used (PDB code)	3V57 Chimera*	3V57 Chimera*	3V57 Chimera*	3V57**
Model resolution (Å)				
FSC threshold = 0.5	3.51	3.27 (4.31~3.19⁺)	5.9	6.11 (3.38⁺⁺)
Map sharpening B factor ($Å^2$)	N/A	N/A	N/A	N/A
Model composition				
Non-hydrogen atoms	3,710	5,735	3,500	1,406
Protein residues	469	721	435	172
Ligands	PEB^: 6	PEB^: 9	PEB^: 6	PEB^: 3
B factors ($Å^2$)				
Protein	51.31	85.27	124.99	21.68
Ligand	52.29	86.84	151.10	22.50
R.m.s. deviations				
Bond lengths (Å)	0.015	0.018	0.023	0.026
Bond angles (°)	0.950	1.461	2.049	2.237
Validation				
MolProbity score	0.93	1.11	1.12	1.55
Clashscore	1.76	3.15	3.3	10.71
Poor rotamers (%)	0.27	0.71	0.29	0.00
Ramachandran plot				
Favoured (%)	98.47	98.14	98.11	99.39
Allowed (%)	1.53	1.57	1.89	0.61
Disallowed (%)	0	0.29	0	0

*Chimeric model from 3V57 and 4LMX detailed in Methods
**Truncated 3V57 chain B detailed in Methods
⁺Resolution range where the high resolution is quoted for chains FC and HC (Ma *et al.* naming) along with their CALM domains and the lower resolution is quoted for chain GC and the associated CALM with a global resolution also calculated
⁺⁺High resolution is for the modelled portion of Linker 3 only (without the associated PE β with poor, possibly partially occupied, density)
^PEB: Phycoerythrobilin

Supplementary Table 1. Cryo-EM refinement and validation statistics
Note that RMS deviations are inflated due to statistics for the PEB chromophores.

Complex	Chain name	Ma *et al.* Equivalent	Protein Component
**L_R6: PE β**	A	b4	L_R6*
PDB *7LIZ*	B	Z4	PE β
	C	a4	PE β
**CaRSP1: PE β**	A	—	CaRSP1
PDB *7LIX*	B	HC	PE β
	C	FC	PE β
	D	GC	PE β
**CaRSP2: PE β**	A	—	CaRSP2
PDB *7LIY*	B	AC	PE β
	C	CC	PE β
**Linker 3: PE β**	A	o4	Linker 3**
PDB *7LJ0*	B	BC	PE β

*Only Pfam00427 and CALM1 modeled in Ma *et al.* [3].
**Only Pfam00427 modeled in Ma *et al.* [3].

Supplementary Table 2. Chain names in deposited crystal structures
Correspondence of chain names between structures in this work and subunits in Ma *et al.* [3].

Supplementary Note 1

Examination of non-CALM bound 'lone' PE β subunits

Six of the 'lone' PE β subunits (those not partnered to PE α as part of a PBS-αβ protomer) that are conserved in both PBS cryo-EM structures are not bound to a CALM domain. To stabilise them, they either bind to alternative partner proteins or they appear to have their N-terminal domain truncated.

In the pair of symmetry mates that adopt the third, extended metamorphic state for hX and hY (*P. purpureum* 6KGX chains IC and I5—I5 visible in Fig. 1a; *G. pacifica* 5Y6P chains iH and iG—the latter visible in Supplementary Fig. 1a), the N-terminal domain of the PE β chain binds to three rod core linker proteins, anchoring the PE β structure (Supplementary Fig. 8c, 9d); $L_{RC}3$ which glances the PE β structure (Supplementary Fig. 9d, orange), $L_{RC}2$ which binds the patch that would be covered by the region of a CALM domain exiting the β-ribbon (Supplementary Fig. 9d, green) and $L_{RC}6$ where the PE β N-terminal domain buries its hydrophobic surface by wrapping around the $L_{RC}6$ FAS1 domain (Supplementary Fig. 9d, red).

A second pair of non-CALM bound 'lone' PE β subunits appears to be N-terminally truncated, with no EM map density for helices hX or hY even though there appears to be room for these helices (*P. purpureum* 6KGX chains LC and L5 with L5 visible in Fig. 1a and Supplementary Fig. 8c, gold cartoon; *G. pacifica* 5Y6P chains iF and iE with iE visible in Supplementary Fig. 1a). An unstructured loop of rod linker L_R9 is positioned on the hydrophobic surface that would usually be covered by the region of a CALM domain exiting the β-ribbon (Supplementary Fig. 9e). Thus, rod linker L_R9 appears to scaffold this PE β subunit. It is possible that helices hX and hY have been proteolytically cleaved *in vivo*, as such processing has been observed in cyanobacteria [4].

The last pair of non-CALM bound 'lone' PE β subunits (*P. purpureum* 6KGX chains B5 and BC, the latter visible Fig. 1a; *G. pacifica* 5Y6P chains kQ and kR, the latter visible Supplementary Fig. 1a) has particularly weak EM map density and they appear to have no EM map density for helices hX and hY. Examining the structure, we observed that helices hX and hY are unlikely to be present; if they adopt the PBS ring conformation, then they would overlap with hexamer Hc' (or symmetry Hc), while if they adopt the cryptophyte conformation they would overlap with "Linker 3" (see Supplementary Note 11). Thus, it appears that the N-terminal domain comprising helices hX and hY has also been proteolytically cleaved for these PE β subunits.

Supplementary Note 2

Metamorphosis of PE β

We analysed the metamorphic transition in PE β by comparing the structure of the N-terminal domain of the PBS ring fold (Fig. 1e) to the CALM-bound fold (Fig. 1f). The transition involves a hinged rotation about the junction between the N-terminal domain and the globin fold domain, where Ile31-Ala32 unwind from helix hY of the PBS form to create the hinge. In the PBS form, the residues between helices hX and hY (Lys15-Gly21) form an extended chain. This extended chain positions Tyr18 so that its side chain slots between helix hE and the junction between helices hA and hB in the PE α subunit to form the stable αβ protomer.

A similar interaction is made by Phe18 of PE α, which slots into the equivalent groove in PE β. Upon metamorphosis to the CALM-bound form, Ala16 to Gly20 adopt a regular β structure (as opposed to an extended chain), which is facilitated by Asn11 to Ala14 unwinding from the C-terminus of helix hX and Gly21 unwinding from the N-terminus of helix hY.

There are a few subtle variants of the cryptophyte form seen in the 'lone' PE β subunits of the two PBS structures, mainly regarding the orientation of helix hX and the length of the β strand (Supplementary Fig. 8a-c). The metamorphic nature of this region of the PE β subunit is reinforced by the fact that for each of the two PBSs studied in this work, a single sequence adopts these two distinct folds, as there is only a single gene for PE β. This is further highlighted by one of the non-CALM bound 'lone' PE β subunits, 6KGX chain I5 (and symmetry mate chain IC), in which the N-terminal domain adopts a third structure where helices hX and hY take on an extended loop conformation upon binding to (and wrapping around) the FAS1 domain of $L_{RC}6$ (Supplementary Fig. 8c, green (I5)).

Supplementary Note 3

Rotamer changes in PE β to accommodate CALM domain binding

As discussed in the main text and above, the CALM-bound PE β subunits and the cryptophyte β subunits share a common structure due to the metamorphic N-terminal domain (Fig. 3g-h, Supplementary Fig. 8c). Another commonality between CALM bound PE β subunits and cryptophyte β subunits is a series of rotameric rearrangements of conserved residues along one face of helix hE when compared with PE β structures in the PBS ring conformation. Helix hE constitutes a large part of the hydrophobic interface across which CALMs and cryptophyte α subunits bind. Most prominent among the observed rotamer rearrangements are Tyr95 and Arg91 (Supplementary Fig. 8d), which make way for the conserved proline at the start of β strand S1 as seen in all CALM domains and cryptophyte α subunits (Fig. 1g, Supplementary Fig. 3). These residues lie on successive turns of helix hE. After β strand S2, the CALM domain again crosses helix hE, including the final conserved hydrophobic residue (Fig. 2d, Supplementary Fig. 3). To accommodate this hydrophobic interaction, the side chain of Arg84 adopts a different rotamer to that observed in PBS ring structures (Supplementary Fig. 8d). Arg84 lies on the same face of helix hE, preceding Arg91 by two turns of the helix. We note that, apart from these three exceptions, essentially all non-surface residues in PE β subunits adopt identical rotamer conformations, whether they are in the PBS ring conformation or the CALM-bound/cryptophyte conformation.

Supplementary Note 4

CALM aromatic motif

The first part of the CALM motif is largely aromatic (Fig. 2d, Supplementary Fig. 3) and is not conserved in cryptophyte α subunits nor is it present in the first CALM repeat of both the *P. purpureum* PBS rod linker L_R6 and the triple CALM protein, CaRSP1 (Fig. 2c). Structures of the aromatic segment of the second CALM repeats from L_R6 and CaRSP1 make it clear that the initial segment often forms a short (sometimes distorted) single turn helix that is terminated by the conserved proline. Along with the conserved asparagine, the motif cradles pyrrole ring D of the PE β chromophore β82. In cryptophyte light harvesting protein structures, the N-termini of genuine cryptophyte α subunits make a similar contact with

pyrrole ring D of PE β82 chromophore [5-7] while in the PBS, a similar contact is often made by the rod linker proteins sitting in centre of the PBS hexamers.

Supplementary Note 5

Classification and evolution of CALM domain containing proteins

CALM domain containing proteins have two obvious phylogenetic groupings; the cryptophyte α subunits containing a single CALM and the CaRSPs of red algae, which usually contain multiple CALM domains. Our results show that the CaRSPs can be further broken down into subgroups. The first subgroup comprises the rod linker L_R6 family (or "Linker 2" family of Lee *et al.* [2]), for which there are currently three members: *P. purpureum* L_R6, which contains two C-terminal CALM domains; and the two "Linker 2"/ L_R6 proteins from *Porphyra umbilicalis* and *Gracilariopsis chorda* [2], which, according to sequence homology, each contain a single C-terminal CALM domain (Supplementary Fig. 2, 3). A second subgroup is the CaRSP1 family, of which we can identify three sequences out of the 13 sequences identified in this work, three belong to this subfamily (Supplementary Fig. 2, 3). The CaRSP1 family members are identified by an F-W-K-[TS] consensus sequence that appears after the second CALM domain (Supplementary Fig. 2, 3). In the structure of CaRSP1 from *P. purpureum,* this F-W-K-S sequence forms a structural motif that anchors CaRSP1 to a PE α subunit in rod c' (specifically, ring Rc'2II) (Supplementary Fig. 4d). Although we do not have any sequence data for *G. pacifica*, superposing *P. purpureum* CaRSP1 onto its structurally homologous CaRSP in the *G. pacifica* structure shows weakly resolved map density that corresponds to this sequence, where the motif plays the same anchoring role (Supplementary Fig. 4e). CALM2 of the putative CaRSP1 from *G. pacifica* also has a tight turn between the two β -strands of the CALM β-ribbon, as seen in *P. purpureum.* The last identified subgroup is CaRSP2, which is identified by its one or two copies of a distinct N-terminal GP-rich motif. Three members of this family have been identified amongst the 13 CaRSP sequences (Supplementary Fig. 2). Finally, the four remaining CaRSP sequences remain unclassified.

Although the PE β subunits positioned by L_R6 and CaRSP1 in *P. purpureum* have precise correlates in the *G. pacifica* PBS structure, the CaRSPs that anchor these PE β subunits in *G. pacifica* must be distinct from those in *P. purpureum*. In *G. pacifica*, L_R6 is replaced by $L_{R\gamma}6$ which is unrelated and does not coordinate PE β subunits, thus the CaRSP that localizes the equivalent PE β subunits must belong to a distinct family of CaRSPs (i.e. not the L_R6 family, as it does not have an N-terminal Pfam00427 PBS linker domain) even though it follows the same path as L_R6 in *P. purpureum*. This suggests that these two proteins have arisen via recombination of the CaRSP domain with two different anchoring domains (PBS linker for L_R6 in *P. purpureum* and an unidentified anchor for *G. pacifica*). In *G. pacifica* CaRSP1, there is no trace of an N-terminal anchoring helix (as seen in the *P. purpureum* CaRSP1, where it has the best map density due to being buried in a PBS rod structure; Fig. 2c, Supplementary Fig. 4c), suggesting that this CaRSP1 is also distinct from that in *P. purpureum*. These observations attest to a high degree of recombination and gene duplication occurring in the CaRSP and PBS linker families in the red algal nucleus.
From extensive BLAST searches, all of these CALM domain-containing proteins come from either mesophilic red algae or cryptophytes. Given that the cryptophytes evolved via secondary endosymbiosis of an ancestral red alga, this suggests that the CALM domain containing proteins evolved in red algae before the secondary endosymbiosis of a red algal organism that resulted in the emergence of the cryptophytes.

Supplementary Note 6

Structure of the C-terminal domain of the rod linker L_R6 in P. purpureum and the associated PE β subunits

Examination of the cryo-EM map for the first CALM domain of the rod linker L_R6 indicated that there was a two-residue frame shift starting in β strand S2 in the model as deposited as PDB 6KGX [3]. The frame shift was probably due to a number of factors including: the ambiguous density at the turn between the two β strands and the presence of small (often β branched) residues in β strand S2. Increasing the number of residues in the loop between the β strands by two produced a frame shift of two residues in the remainder of the CALM domain (Fig. 1g). This resulted in improving the overall stereochemistry of the model and a better fit to the EM map density, particularly residues His344 and Tyr346 in β strand S2 (compare the fit for the revised model (Supplementary Fig. 4a) versus the original model (Supplementary Fig. 4b)), and Tyr353 at the end of β strand S2 (not shown). The frame shift also resulted in a better structure-based alignment of this CALM domain to the cryptophyte-α subunit structures and matched the CALM motifs as discovered via the program MEME [8]. The remaining residues of the L_R6 sequence were traced through to the second CALM domain. Although the EM map density for the bridge between the two CALM domains was weak, we were able to determine the register of the sequence using the conserved structural position of the N-x-A-P motif in the second CALM domain. In addition, we completed the models of the associated PE β subunits by building the cryptophyte-like N-terminal domains comprising helices hX and hY plus the intervening β strand that forms a β sheet with the CALM domain β hairpin.

The final model for the C-terminal domain of rod linker L_R6 in *P. purpureum* comprises two CALM domains connected by a linker (residues Gly309 through to Lys423) and it anchors two PE β subunits (chains Z4 and a4, Fig. 2a; with a symmetry-related structure anchoring symmetry mates Z1 and a1) to the Hd' lone hexamer (and Hd symmetry mate) via the N-terminal PBS linker domain (Pfam00427; Fig. 1a). The two PE β subunits linked by the two CALM domains of L_R6 form a globular structure (Fig. 2a). The β hairpin of the second CALM domain lies between the two PE β subunits, stabilising the pair into one compact unit. In each CALM domain of L_R6, the highly conserved asparagine residue (N-x-A-P) interacts with pyrrole ring D from the β82 chromophore in the associated PE β subunit. Specifically, the asparagine makes a hydrogen bond between the side chain carbonyl oxygen and pyrrole (ring D) nitrogen plus a backbone amide to pyrrole (ring D) carbonyl hydrogen bond. The second L_R6 CALM domain contains the N-terminal aromatic motif (Fig. 2a). It also interacts with the β82 chromophore in the associated PE β subunit. The first and last aromatic residues in the motif (Trp382 and Tyr386) contact this chromophore, while the intervening aromatic residue (Tyr385) contacts the break between PE β subunit helices hFa and hFb.

Supplementary Note 7

Structure of CaRSP1 from P. purpureum and the associated PE β subunits

CaRSP1 starts with an α helical structure that penetrates between the two innermost hexameric ring structures in Rod b' (Rb'1II and Rb'2I; Fig. 2c, Supplementary Fig. 5b). This segment has some of the best map density observed for CaRSPs due to its position in Rod b' (Supplementary Fig. 4c). CaRSP1 then forms a globular structure containing the first

two CALM domains scaffolding two PE β subunits (HC and FC). Density between the CALM domains is weak, with a break between Ala117 and Ala119, inclusive. Density for CALM2 is excellent, including the subsequent linking segment up to Ser176, which ends the F-W-K-S motif.

In CALM1, the asparagine of the N-x-A-P motif is replaced by Ser58. This residue does not interact directly with the β82 chromophore, instead, it makes a side chain hydrogen bond with Arg84 which interacts with the propionate side chain of chromophore β82. The two CALM domains are linked by a short helical segment (Pro105-Thr110) followed by an extended chain (Tyr111–Asn126), where both of these structures contact PBS rods (Supplementary Fig. 5b); the former at the base of Rod g' (Rg'1I), and the latter along Rod b' (Rb'2II). In CALM2, Asn138 (N-x-A-P motif) interacts with the side chain of Arg84 and does not directly interact with the β82 chromophore, as per CALM1 Ser58. CALM2 does not have an α helix, instead, the extended chain leaving the β hairpin forms a second short β hairpin, where the turn in the hairpin contacts the outer hexamer ring of Rod c' (specifically, trimer Rc'3I). This is followed by the most prominent contact containing the F-W-K-S motif that contacts ring Rc'2II on Rod c' (Supplementary Fig. 3, 4d, 5b).

The chain linking the N-terminal globular structure (CALM1, CALM2 plus PE β HC and FC) to the second globular structure (CALM3 plus PE β GC) is weak, with several chain breaks (Fig. 2c). There is clear map density for Asn182-Ala190, due to the hydrophobic contacts made by Tyr183 and Pro186 (see below Supplementary Note 10 on "anchors"). Although there is weak map density between Ala190 and Ala200, we could not model it in an unambiguous fashion.

The map density for the globular structure formed by CaRSP1 CALM3 plus PE β subunit GC was not of the same quality as the two preceding CALM:PE β structures, however, the backbone was unambiguous, and we were able to model CaRSP1 up to Thr253 at terminus of the CALM3 α helix.

Supplementary Note 8

Structure of CaRSP2 from P. purpureum and the associated PE β subunits

In the overall cryo-EM map for *P. purpureum* (EMD-9976) there was no clear map density for the four unique 'lone' PE β subunits AC plus CC (and the two symmetry mates); it is only upon careful inspection of the local maps in these regions (EMD-9978 or EMD-9986) that weak density corresponding to the PE β chains is observed. However, the orientation of the density is not consistent with the coordinates deposited in the PDB (accession code 6KGX). Rigid body fitting in COOT clearly revealed that when both PE β chains (and their symmetry mates) are reoriented, they match the density more clearly. Subsequently, each reoriented PE β allows for the clear identification of density supporting a remodelled N-terminal domain, such that it adopts a cryptophyte-like PE β fold (Fig. 1d). Map density for the two associated CALM domains, belonging to CaRSP2, is also clear (Fig. 1d, 2b). Given the weak EM map density for this region, PE β chains were restrained to a reference model during refinement (see Materials & Methods).

CaRSP2 and its two associated PE β subunits (AC and CC) form a globular structure that is near the membrane interface (Fig. 1a). The two termini of the observed structure of CaRSP2 (Leu194 and Leu276) lie close to the membrane and potentially integral membrane proteins.

No map density is seen beyond these termini. The complex interacts with the inner two hexamers of Rod a' with contacts made by both CaRSP2 and PE β chain CC (Supplementary Fig. 5c). PE β chain AC appears to make no interactions with the PBS other than through CaRSP2 and PE β chain CC, which is attached to the second CALM domain of CaRSP2.

Supplementary Note 9

Features of seven modelled CALM domains

The seven modelled CALM domains show highly conserved structural characteristics, with some deviations (Supplementary Fig. 8a-b); notably, the conserved CALM motif is largely composed of hydrophobic residues (Fig. 2e, Supplementary Fig. 3). Upon close inspection, CALMs without a tight turn between β-strands of the β-ribbon (all but CaRSP1-CALM2 which binds PE β FC) appear to have a short, distorted helical turn leading into the exiting β-strand. This is a feature observed in the closed form cryptophyte α subunits of PE545 [7] and PC645 [5]. Conversely, only three CALM domains appear to have a C-terminal α helix (which is always seen in cryptophytes [5-7]). Six of the modelled CALM domains (CaRSP1 CALM1 being the exception) also appear to have a 4-6 residue single turn helix (Supplementary Fig. 8b, bottom right) that usually coincides with the aromatic motif (Fig. 2e).

Supplementary Note 10

CaRSP proteins anchor PE β subunits to the PBS

Each CaRSP makes multiple contacts to PBS structures, especially in the sequences between CALM domains and at the turn in the CALM β hairpin (Supplementary Fig. 5). These contacts are often mediated by the side chains of aromatic residues. The result is that the CALM-bound PE β subunits are specifically localized on the surfaces of PBS rods.
The complex formed by CaRSP L_R6 and its two attached PE β subunits (Z4 and a4) makes a compact, globular structure (Supplementary Fig. 2a) that is nestled between Rods c and e and Hexamers Hd' and Hc (Fig. 1a). The CaRSP L_R6 is anchored to hexamer Hd' via its N-terminal PBS linker domain (Pfam00427) and then proceeds to the first PE β subunit (Z4), where it contacts the first hexamer of Rod c (specifically, trimer Rc1I) via the side chain of Tyr325 before the start of the β hairpin motif of CALM1 (the first CALM domain; Supplementary Fig. 5a). At the β turn at the apex of the hairpin, Ser341 and His344 contact the edge of hexamer Hd' (Supplementary Fig. 5a). The α helix of CALM1 (residues 357-363) also contacts hexamer Hd'. Ser406 at the turn between the β strands of the second CALM domain contacts a chromophore on hexamer Hc (Supplementary Fig. 5a). Additional contacts are made with Rod e (Re2II) via the second PE β (a4) attached to L_R6 (Gly65, Ser68, Gly70 and Pro123) and to Rod c (Rc1I) via the first PE β (Z4 Ala119).

The complex formed by CaRSP1 and its three associated PE β subunits (HC, FC and GC) forms an extended structure that runs between Rods b' and c' (Fig. 1a). It can be broken into two compact sections (Fig. 2c): the N-terminal segment of CaRSP1 with two CALM domains and two PE β subunits (HC and FC) hugging the surface of Rod c' and contacting the inner ring of Rod g' (Rg'1I); and the final CALM3 domain and its associated PE β (GC) at the outer surface of the PBS between Rods c' and b' (Supplementary Fig. 5b). The N-terminus of CaRSP1 forms an α helix (Fig. 2d) that is anchored in Rod b' penetrating between the inner most hexamer rings (trimers Rb'1II and Rb'2I, Supplementary Fig. 4c and 5b). It then

enters the first CALM domain and at the turn in the β ribbon, Met72 contacts the outer edge of the innermost hexamer of Rod c' (trimer Rc'1II; Supplementary Fig. 5b). The start of the helix in CALM1 (Ala88 and Met91) makes a hydrophobic contact with central hexamer of Rod c' (trimer Rc'2I). After the end of the helix, Met109 and Thr110 contact the base of Rod g' (trimer Rg'1I) before CaRSP1 crosses back to Rod b', where it is anchored to the central hexamer (trimer Rb'2II) by a hydrophobic contact made by the side chain of Tyr120 (Supplementary Fig. 5b). From this point, CaRSP1 enters CALM2, anchoring the second PE β subunit (FC). CALM2 does not have an α helix but exits the β ribbon as an extended chain. This extended chain is terminated by a short β ribbon, where the tight turn makes a hydrophobic contact with a chromophore on the outermost hexamer of Rod c' (trimer Rc'3I) via Asn162 and Gly163. CaRSP1 then turns inwards, anchoring to the central hexamer of Rod c' (trimer Rc'2II) via the F-W-K-S motif, where the aromatic side chains make hydrophobic contacts (Supplementary Fig. 4d). CaRSP1 then follows an extended path along Rod c' where the side chains of Tyr183 and Pro186 make a hydrophobic contact with the outer hexamer of Rod c' (hexamer Rc'3). We note that many of these contacts observed in the *P. purpureum* CaRSP1 structure (apart from the N-terminal helix) are also seen in the cryo-EM map density for the *G. pacifica*.

The C-terminal portion of CaRSP1 forms a globular structure with its CALM3 domain anchoring PE β (GC, Fig. 2c) that sits between Rods c' and b' at the surface of the PBS (Fig. 1a). Residues around the N-terminal aromatic motif of CALM3 make multiple contacts with the outermost hexamer of Rod c' (trimer Rc'3II with CaRSP1 residues: Gln203, Arg204 and Arg209; Supplementary Fig. 5b). Near the turn in the β hairpin, the side chains of Arg220 and Phe224 in β strand S1 contact the outermost hexamer of Rod b' (hexamer Rb'3; Supplementary Fig. 5b).

The portion of the complex between CaRSP2 and its two PE β subunits (CC and AC) that is visible in the cryo-EM map density forms a compact globular structure that is likely to be proximal to the membrane and interacts with Rod a' (Fig. 1a). Tyr219 on β strand S2, just after the turn between the two β strands makes a hydrophobic contact with the innermost hexamer of Rod a' (trimer Ra'1II Supplementary Fig. 5c). In a similar fashion, the residues around the turn between β strands S1 and S2 in CALM2 (Ser252-Thr260) make extensive contacts with the central hexamer in Rod a' (trimer Ra'2I). Finally, Pro274 near the end of CALM2 makes a hydrophobic contact with the innermost hexamer of Rod a' (trimer Ra'1II; Supplementary Fig. 5c).

Supplementary Note 11

Structure of C-terminus of the novel PBS linker protein "Linker 3"

There is a clear, unmodelled β ribbon structure close to the B5/BC 'lone' PE β subunits, but it differs in orientation and location from a CALM domain while still covering much of the remaining hydrophobic surface left following the possible cleavage of helices hX and hY (Supplementary Fig. 9a-c). Given the quality of the map density for this β ribbon structure, we were able to build an atomic model and use identifying side chains to show that the sequence is consistent with the C-terminal domain of "Linker 3" from *P. purpureum* (KAA8497087), which is a new class of linker protein that diverged from L_R1 after transfer to the nucleus in red algae and contains an N-terminal Pfam00427 domain [2]. This β ribbon differs from a CALM domain in that it does not show a characteristic twist at the start of the ribbon (seen as a cross-over of β strands in Fig. 1b) and it does not contain the motivic

features seen in CALM domains (i.e. an "AP" sequence). In the deposited coordinates, this map density sits proximal to the PBS linker domain of a second copy of L_R6 in *P. purpureum* and to L_R2 *G. pacifica*, where in both cases there is no model beyond the PBS linker domain however, there exists very weak map density connecting each PBS linker domain to the observed β ribbon map density. It is likely that in each instance, the PBS anchored Pfam00427 domain is actually that of "Linker 3", which was not known at the time these structural models were generated.

The anchoring pattern seen in Linker 3, where the chain makes multiple contacts with several rods, is similar to that seen for the CaRSPs (Supplementary Fig. 9a). The Pfam00427 domain sits inside hexamer Hc where the chain leading toward the 'lone' PE β subunit BC makes several contacts with hexamer Rc2. The chain stays in contact with trimer Rc2I while it enters the β ribbon. At the turn of the beta ribbon, a hydrophobic contact is made with trimer Rc1II and the exiting beta ribbon makes further contacts with timer Rc2I. The chain can be traced through weak density (although not modelled in the final structure) to cross the void to Rod e where the initial contact made with the rod is a short structural motif reminiscent of CaRSP1's F-W-K-S motif where Phe303 and Trp304 of Linker 3 straddle Tyr83 of PE α on Rod e (Re3II) (Supplementary Fig. 4f); the sequence here however reads F-W-A-K. The density then becomes hard to unambiguously assign and while it was not modelled in the final structure, however, the chain can be traced to run down into the groove between trimers Re3II and Re4I where the density terminates on Re4I (Supplementary Fig. 9a).

Supplementary Note 12

Evolutionary timing of the emergence of the CaRSPs

We note that the CaRSPs we have identified (including L_R6/"Linker 2") span the two subphyla of mesophilic red algae [9], with no CaRSPs identified (so far) in the thermoacidophilic *Cyanidiophytina* subphylum. This pattern matches the diversification of the L_R1 linker family [2], indicating that both the expansion of the L_R1 linker family and the evolution of the CALM containing CaRSPs (including L_R6/"Linker 2") occurred early in red algal evolution [9].

Supplementary Note 13

Alternative scenario for the emergence of the cryptophyte light harvesting antenna

An alternative scenario is that the demise of the PBS and establishment of the cryptophyte light harvesting antenna via the mechanism described in the main text occurred in a red algal species prior to secondary endosymbiosis, where this new red alga became the endosymbiont. Furthermore, massive gene loss events have been observed in red algae [10] and similar events may have destabilised the PBS to make way for a cryptophyte-like antenna in an ancient red alga. To date, no red algae have been observed with a cryptophyte-like system, however, this alternative model cannot be ruled out. We also note that competition between PE α and CALM domains does not appear to be a problem for extant red algae.

Supplementary Note 14

Why have CaRSPs bound only to PE β subunits?

There are some subtleties with the assignment of 'lone', globin-folded proteins as PE β subunits. In both deposited cryo-EM structures [1,3] all 'lone' proteins are modelled as PE β subunits due to characteristic residue differences; however, this is not without some ambiguity. Also, given the low sequence identity between CALM domains, one would assume that CALM domains are likely to be promiscuous in their binding behaviours. Firstly, PBS PE α subunits can be ruled out as likely binding partners for CALM domains given: their alterations in the orientation of helices hG and hH; the removal of two chromophores with relation to PBS PE β; and low sequence identity. This is the same for PC α. Additionally, one of these missing chromophores sits just under the loop between the β strands of the CALM domain and thus may interfere with the interaction with a CALM domain.

For PBS PC β, something more confounding may be happening. Given that the ratio of PE:PC is approximately 7.3:1 in the *P. purpureum* PBS (and even larger in *G. pacifica*), any statistical mixture of PE β with PC β in the 'lone' β subunits would be hard to identify as the map density would be overwhelmed by signal from PE β. There are however characteristic differences between PE β and PC β, like the changes to the loop between helices hG and hH and chromophore alterations, but these again may not be resolved easily if there is a statistical mixture, and the sequence similarity is much higher than that of PBS α subunits with PE β.

A physical argument can be made for why PE β is chosen over PC β; that CaRSPs are scaffolding phycobiliproteins to the more distal regions of the PBS rod structures where the rods are more blue absorbing (i.e. the principal chromophores are phycoerythrobilins). If the CaRSP-bound β subunits are PC β rather than PE β, this would result in a net loss of excitation energy from rod system and thus, the photosystem. However, given that the ratio of PE:PC is approximately 7.3:1, this may not matter to the organism. Furthermore, despite nomenclature, the β subunits of all cryptophytes have evolved from the red algal PE β subunit [11], which suggests that either PE β has always been the binding partner of the CALM domain or PE β won out over PC β following secondary endosymbiosis.

The question posed here amounts to asking why PE β subunits do not bind to PC α in the PBS (or indeed PC β to PE α). Sequences of PC β and PE β in *P. purpureum* are identical along the hydrophobic surface to which a CALM would bind (or the cognate PBS α). There are some sequence differences in the N-terminal helices hX and hY, including in the metamorphic region that switches to β-strand in the cryptophyte-like conformation, however, comparison between the PC β and PE β sequences in this region gives no obvious suggestion that PC β is not metamorphic and thus cannot adopt the cryptophyte-like fold and bind a CALM domain. It may be that metamorphosis or subtle residue alterations play a key role here for molecular recognition and may be the cause of the CALM-binding ability of PE β rather than PC β, but this remains unclear.

1 Zhang, J. *et al.* Structure of phycobilisome from the red alga Griffithsia pacifica. *Nature* **551**, 57-63, doi:10.1038/nature24278 (2017).
2 Lee, J., Kim, D., Bhattacharya, D. & Yoon, H. S. Expansion of phycobilisome linker gene families in mesophilic red algae. *Nat Commun* **10**, 4823, doi:10.1038/s41467-019-12779-1 (2019).
3 Ma, J. *et al.* Structural basis of energy transfer in Porphyridium purpureum phycobilisome. *Nature* **579**, 146-151, doi:10.1038/s41586-020-2020-7 (2020).
4 Soni, B. R. *et al.* Structure of the novel 14kDa fragment of alpha-subunit of phycoerythrin from the starving cyanobacterium Phormidium tenue. *J Struct Biol* **171**, 247-255, doi:10.1016/j.jsb.2010.05.008 (2010).
5 Harrop, S. J. *et al.* Single-residue insertion switches the quaternary structure and exciton states of cryptophyte light-harvesting proteins. *Proc Natl Acad Sci U S A* **111**, E2666-2675, doi:10.1073/pnas.1402538111 (2014).
6 Doust, A. B. *et al.* Developing a structure-function model for the cryptophyte phycoerythrin 545 using ultrahigh resolution crystallography and ultrafast laser spectroscopy. *J Mol Biol* **344**, 135-153 (2004).
7 Wilk, K. E. *et al.* Evolution of a light-harvesting protein by addition of new subunits and rearrangement of conserved elements: crystal structure of a cryptophyte phycoerythrin at 1.63-A resolution. *Proc Natl Acad Sci U S A* **96**, 8901-8906 (1999).
8 Bailey, T. L. *et al.* MEME SUITE: tools for motif discovery and searching. *Nucleic Acids Res* **37**, W202-208, doi:10.1093/nar/gkp335 (2009).
9 Munoz-Gomez, S. A. *et al.* The New Red Algal Subphylum Proteorhodophytina Comprises the Largest and Most Divergent Plastid Genomes Known. *Curr Biol* **27**, 1677-1684 e1674, doi:10.1016/j.cub.2017.04.054 (2017).
10 Qiu, H., Price, D. C., Yang, E. C., Yoon, H. S. & Bhattacharya, D. Evidence of ancient genome reduction in red algae (Rhodophyta). *J Phycol* **51**, 624-636, doi:10.1111/jpy.12294 (2015).
11 Apt, K. E., Collier, J. L. & Grossman, A. R. Evolution of the phycobiliproteins. *J Mol Biol* **248**, 79-96 (1995).
