# Supplementary material for: Scaffolding proteins guide the evolution of algal light harvesting antennas
Source: Nat Commun. 2021 Mar 25;12:1890. doi: 10.1038/s41467-021-22128-w (PMC7994580; doi:10.1038/s41467-021-22128-w)
Supplement: Supplementary file 3 — Reporting Summary [file 41467_2021_22128_MOESM3_ESM.pdf]

nature research

Corresponding author(s): Paul Curmi
Last updated by author(s): Jan 28, 2021

Reporting Summary

Nature Research wishes to improve the reproducibility of the work that we publish. This form provides structure for consistency and transparency in reporting. For further information on Nature Research policies, see our Editorial Policies and the Editorial Policy Checklist.

Statistics

For all statistical analyses, confirm that the following items are present in the figure legend, table legend, main text, or Methods section.

n/a	Confirmed	
☒	☐	The exact sample size (*n*) for each experimental group/condition, given as a discrete number and unit of measurement
☒	☐	A statement on whether measurements were taken from distinct samples or whether the same sample was measured repeatedly
☒	☐	The statistical test(s) used AND whether they are one- or two-sided<br>*Only common tests should be described solely by name; describe more complex techniques in the Methods section.*
☒	☐	A description of all covariates tested
☒	☐	A description of any assumptions or corrections, such as tests of normality and adjustment for multiple comparisons
☒	☐	A full description of the statistical parameters including central tendency (e.g. means) or other basic estimates (e.g. regression coefficient) AND variation (e.g. standard deviation) or associated estimates of uncertainty (e.g. confidence intervals)
☒	☐	For null hypothesis testing, the test statistic (e.g. *F*, *t*, *r*) with confidence intervals, effect sizes, degrees of freedom and *P* value noted<br>*Give P values as exact values whenever suitable.*
☒	☐	For Bayesian analysis, information on the choice of priors and Markov chain Monte Carlo settings
☒	☐	For hierarchical and complex designs, identification of the appropriate level for tests and full reporting of outcomes
☒	☐	Estimates of effect sizes (e.g. Cohen's *d*, Pearson's *r*), indicating how they were calculated

Our web collection on statistics for biologists contains articles on many of the points above.

Software and code

Policy information about availability of computer code

Data collection	No software was used for data collection.
Data analysis	ChimeraX 1.1; ISOLDE 1.1; PyMol 2.3.4; Phenix 1.18; Coot 0.8.9.2; MEME; Mathematica 12.0.0.0; PsiBLAST; PhiBLAST; MAFFT; ClustalW; MUSCLE

For manuscripts utilizing custom algorithms or software that are central to the research but not yet described in published literature, software must be made available to editors and reviewers. We strongly encourage code deposition in a community repository (e.g. GitHub). See the Nature Research guidelines for submitting code & software for further information.

Data

Policy information about availability of data

All manuscripts must include a data availability statement. This statement should provide the following information, where applicable:
- Accession codes, unique identifiers, or web links for publicly available datasets
- A list of figures that have associated raw data
- A description of any restrictions on data availability

Atomic coordinates for models generated in this work have been deposited with the Protein Data Bank with accession codes for: CaRSP1 plus associated PE beta subunits as 7LIX [https://www.rcsb.org/structure/7LIX]; CaRSP2 plus associated PE beta subunits as 7LIY [https://www.rcsb.org/structure/7LIY]; LR6 plus associated PE beta subunits as 7LIZ [https://www.rcsb.org/structure/7LIZ]; and Linker 3 plus associated PE beta subunit as 7LJ0 [https://www.rcsb.org/structure/7LJ0]. Cryo-EM maps used in this work were downloaded from the Electron Microscopy Databank with accession codes EMD-9976 [https://www.emdataresource.org/EMD-9976] through to EMD-9988 [https://www.emdataresource.org/EMD-9988] for Porphyridium purpureum (with associated model PDB 6KGX [https://www.rcsb.org/structure/6KGX]) and EMD-6758 [https://www.emdataresource.org/EMD-6758] through to EMD-6769 [https://www.emdataresource.org/EMD-6769] for Griffithsia pacifica (with associated model PDB 5Y6P [https://www.rcsb.org/structure/5Y6P]). Models were built using publicly available structural

models: PDB 4LMX [https://www.rcsb.org/structure/4LMX] and PDB 3V57 [https://www.rcsb.org/structure/3V57]. Accession codes for protein sequences used in this work are KAA8497087 [https://www.ncbi.nlm.nih.gov/protein/KAA8497087] for Linker 3 and KAA8491180 [https://www.ncbi.nlm.nih.gov/protein/KAA8491180], KAA8495560 [https://www.ncbi.nlm.nih.gov/protein/KAA8495560], KAA8491883 [https://www.ncbi.nlm.nih.gov/protein/KAA8491883], XP_005716950 [https://www.ncbi.nlm.nih.gov/protein/XP_005716950], XP_005711070 [https://www.ncbi.nlm.nih.gov/protein/XP_005711070], PXF45458 [https://www.ncbi.nlm.nih.gov/protein/PXF45458], PXF49306 [https://www.ncbi.nlm.nih.gov/protein/PXF49306], OSX75119 [https://www.ncbi.nlm.nih.gov/protein/OSX75119], OSX70945 [https://www.ncbi.nlm.nih.gov/protein/OSX70945], OSX69271 [https://www.ncbi.nlm.nih.gov/protein/OSX69271], OSX70368 [https://www.ncbi.nlm.nih.gov/protein/OSX70368], OSX77362 [https://www.ncbi.nlm.nih.gov/protein/OSX77362], OSX68985 [https://www.ncbi.nlm.nih.gov/protein/OSX68985] for CaRSPs as shown in Extended Data Fig. 2. Publicly available protein structures used for structure-based sequence alignment (Fig. 1) were PDB: 1XF6 [https://www.rcsb.org/structure/1XF6], 4LMS [https://www.rcsb.org/structure/4LMS], 4LMX [https://www.rcsb.org/structure/4LMX], 4LM6 [https://www.rcsb.org/structure/4LM6]. All other data and materials are available from the corresponding author on reasonable request.

Field-specific reporting

Please select the one below that is the best fit for your research. If you are not sure, read the appropriate sections before making your selection.

[x] Life sciences   [ ] Behavioural & social sciences   [ ] Ecological, evolutionary & environmental sciences

For a reference copy of the document with all sections, see nature.com/documents/nr-reporting-summary-flat.pdf

Life sciences study design

All studies must disclose on these points even when the disclosure is negative.

Sample size	All cryo-EM maps deposited for red algal phycobilisome single particle reconstructions for Porphyridium purpureum at 2.82Å resolution were analyzed (overall map EMD-9976 and associated submaps EMD-9977 through to EMD-9988). These were the only maps at sufficient resolution for atomic model building.
Data exclusions	No data were excluded.
Replication	Multiple rounds of structural refinement have been performed with different refinement packages (Phenix and ISOLDE), each resulting in indistinguishable final atomic models on convergence. Regions of the protein where no clear consensus structure could be obtained due to poor map quality were not included in the final atomic models deposited with the PDB.
Randomization	Randomization of samples is not relevant because we are studying specific protein complexes.
Blinding	Blinding is not relevant because we are studying specific protein complexes.

Reporting for specific materials, systems and methods

We require information from authors about some types of materials, experimental systems and methods used in many studies. Here, indicate whether each material, system or method listed is relevant to your study. If you are not sure if a list item applies to your research, read the appropriate section before selecting a response.

Materials & experimental systems

n/a	Involved in the study	
[x]	[ ]	Antibodies
[x]	[ ]	Eukaryotic cell lines
[x]	[ ]	Palaeontology and archaeology
[x]	[ ]	Animals and other organisms
[x]	[ ]	Human research participants
[x]	[ ]	Clinical data
[x]	[ ]	Dual use research of concern

Methods

n/a	Involved in the study	
[x]	[ ]	ChIP-seq
[x]	[ ]	Flow cytometry
[x]	[ ]	MRI-based neuroimaging
